# Seeing Double: A Rare Encounter with a Duplicate Vas Deferens During Total Extraperitoneal Herniorrhaphy, a Case Report and Review of the Literature

**DOI:** 10.51894/001c.168175

**Published:** 2026-08-28

**Authors:** Ahmad Baghdadi, Joseph Azar, Allison Kowalski, Calleb Alley, Bilal Kharbutli

**Affiliations:** 1 Department of General Surgery, Henry Ford Wyandotte Hospital, 2333 Biddle Ave, Wyandotte, MI 48192

**Keywords:** Duplicate vas deferens, anatomical anomaly, inguinal hernia, TEP herniorrhaphy, laparoscopic surgery

## Abstract

**BACKGROUND:**

Asymptomatic anatomical anomalies are often found incidentally during unrelated surgical procedures. One rare congenital anomaly, with an incidence of approximately 0.05%, is duplication of the vas deferens within a single spermatic cord, typically identified during hernia repairs or various urological procedures.

**CASE PRESENTATION:**

We present the case of a 55-year-old male who underwent a total extraperitoneal laparoscopic herniorrhaphy for a right inguinal hernia. Intraoperatively, two vasa deferentia were identified traversing the deep inguinal ring adjacent to each other. The operation was completed with caution to preserve these structures. The patient had an uncomplicated post-operative course.

**CONCLUSION:**

This case underscores the importance of meticulous anatomic identification during inguinal hernia repair. Clinical awareness of such anomalies is essential for avoiding potential surgical complications, including iatrogenic damage to the vas deferens, chronic pain, hematoma, and fistula formation, during inguinal or other urological repairs. This rare case adds to the limited literature on in-surgery recognition of vas deferens duplication.

## INTRODUCTION

The vas deferens is a paired duct within the spermatic cord responsible for transporting sperm from the epididymis to the ejaculatory ducts. Common anomalies of the vas deferens include congenital absence, strictures, and ectopia. These variants may cause symptoms such as odynorgasmia or infertility.^1^ Duplicate vas deferens is a rare and asymptomatic anomaly with an estimated incidence of 0.05%, most frequently identified during hernia repairs or urological procedures such as vasectomy, varicocelectomy and orchidopexy.^1–4^ In the literature, between 1929 and 2022, only 47 cases were reported.^4^ Some recent case reports have also been published.^5,6^ The detection of this anomaly is limited because most patients remain asymptomatic, leading to under-recognition in clinical practice and subsequent under reporting in the literature. This generally asymptomatic anomaly often goes undiagnosed unless encountered intraoperatively. While most reported cases involve unilateral duplication, bilateral duplication has been described only in isolated case reports, with the majority seen in pediatric populations.^7–11^ This highlights the extreme rarity of bilateral involvement and the importance of distinguishing true duplication from other mesonephric remnants or ectopic ureters during minimally invasive herniorrhaphy.

## CASE REPORT

**Figure 1.**
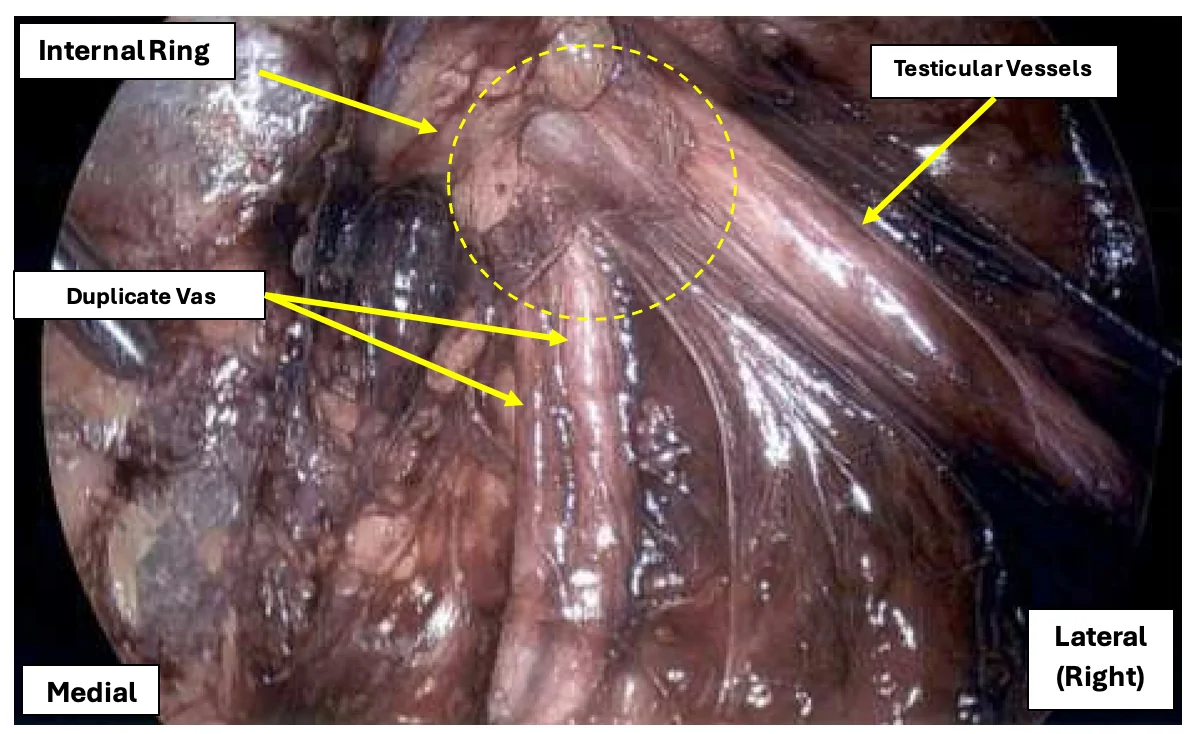
Laparoscopic view of a right inguinal hernia repair during the dissection of the sac and identification of cord structures. The duplicate vas deferens is isolated in a single spermatic cord.

A 55-year-old male presented to our surgical clinic with right inguinal swelling and discomfort that began one month earlier following heavy lifting. On physical examination, a small, reducible right inguinal hernia was palpated with moderate tenderness. Both testes appeared normal on examination. Ultrasound of the right groin revealed laxity of the inguinal ligament and approximately 3 cm of bowel within the hernia. The patient wished to proceed with surgical intervention and consent was obtained. A total extraperitoneal right herniorrhaphy was conducted under general anesthesia. Intraoperatively, a small to moderately sized indirect hernia sac was identified and reduced, along with a moderate sized cord lipoma. Careful identification of the spermatic cord contents revealed a duplicated vas deferens, with both structures traversing the deep inguinal ring adjacent to each other in the expected anatomical location of the vas deferens (**Figure 1**).

All cord components were meticulously identified and preserved. A large 3D Dextile™ Max mesh was then placed on the right side and secured above the pubic bone over the midline using 2 AbsorbaTacks™, ensuring adequate coverage of all potential hernia defects with the hernia sac lying within the cup of the mesh. The surgery was completed without apparent complications, and the patient was discharged the same day. At the postoperative follow-up visit, no concerns were reported and appropriate post-operative recovery was noted. The patient was informed of the anatomical variant and operative findings in detail.

## DISCUSSION

From an embryological perspective, duplication of the vas deferens is a rare congenital anomaly occurring between the 4th and 12th weeks of gestation due to aberrations in mesonephric (Wolffian) duct development. This duct gives rise to the vas deferens, epididymis, and seminal vesicles.^1^ While these embryological aberrations remain incompletely understood, the literature suggests that vas deferens duplication may be the result of fetal mesonephric system duplication or division of the Wolffian duct during organogenesis.^2,3^

A thorough understanding of surgical anatomy is crucial to avoid iatrogenic injury, particularly when aberrant structures are present. Intraoperative recognition of duplicated vas deferens is essential, as accidental trauma to the vas deferens may result in infertility, chronic pain, hematoma or fistula formation as demonstrated by evidence from experimental models^12–14^ as well as case reports from vasectomy procedures.^15–18^ Because this anomaly is typically asymptomatic, surgeons must remain vigilant and perform meticulous dissection when operating on the spermatic cord. In our case, a total extraperitoneal approach was conducted in the hands of an experienced surgeon. It is worth noting that the choice of herniorrhaphy approach and surgeon experience may have different implications on adequate anatomical identification. For instance, multiple large studies have compared different aspects of transabdominal preperitoneal patch plasty (TAPP) to total extraperitoneal patch plasty (TEP).^19–21^ The general current understanding is that TAPP provides a more familiar, wide-angle intra-abdominal view that could offer a better visualization of the vas deferens and cord structures, especially for new learners. In contrast to that, TEP offers a direct visualization of the preperitoneal cavity, but the cord structures may be obscured by a collapsing space in the setting of a smaller and restricted field.

The true incidence of this anomaly may be higher than reported, given the lack of symptoms and reliance on incidental surgical findings. While most reported cases involve unilateral duplication, surgeons should be aware that bilateral duplication, although extremely rare, is possible and may present unexpectedly during surgery.^7–11^ Awareness of bilateral anomalies highlights the need for meticulous dissection and identification of spermatic cord structures. In our case, the left side was not dissected given that we chose the TEP approach, and this may be seen as a limitation of such approaches when compared to TAP.

Careful technique, irrespective of the chosen herniorrhaphy approach can still minimize iatrogenic injury. Moreover, some surgeons may opt to utilize Doppler ultrasonography to confirm the vas deferens duplication by noting the absence of waveforms in these structures, differentiating them from adjacent vessels.^22^

## LIMITATIONS

Our report has several limitations. As an isolated case report, our findings cannot be generalized to determine the true prevalence or clinical patterns of a duplicate vas deferens. Furthermore, advanced preoperative pelvic imaging was not clinically indicated for a routine elective hernia, precluding prospective identification of this anomaly. Finally, given the chosen TEP approach and in the interest of patient safety, the contralateral spermatic cord was not dissected beyond the operative field, leaving the potential bilaterality of the variation unknown.

## CONCLUSION

Duplicate vas deferens is an extremely rare and often asymptomatic congenital anomaly. Identifying such anomalies during herniorrhaphy is critical to maintaining surgical safety; failure to do so may compromise clinical outcomes and expose the surgeon to substantial medicolegal ramifications. This case reinforces the idea that surgeons should maintain a high index of suspicion for aberrant anatomy, perform meticulous dissection, and carefully identify all structures within the spermatic cord. Thorough preoperative planning and intraoperative vigilance should be exercised irrespective of the chosen herniorrhaphy approach. We hereby present a case that supplements the scarce literature on intraoperative recognition of vas deferens duplication, thereby providing a valuable reference for future surgical practice

### PATIENT CONSENT

Written informed consent was obtained from the patient for publication of this case report and any accompanying images. Proof of consent is available with the authors of this case report.

### CONFLICT OF INTEREST STATEMENT

The authors declare that they have no conflicts of interest related to this work.
